# Sparse Representation-Based Discriminative Metric Learning for Brain MRI Image Retrieval

**DOI:** 10.3389/fnins.2021.829040

**Published:** 2022-01-14

**Authors:** Guohua Zhou, Bing Lu, Xuelong Hu, Tongguang Ni

**Affiliations:** ^1^School of Information Engineering, Changzhou Institute of Industry Technology, Changzhou, China; ^2^School of Computer Science and Artificial Intelligence, Changzhou University, Changzhou, China; ^3^College of Information Engineering, Yangzhou University, Yangzhou, China

**Keywords:** medical image retrieval, magnetic resonance imaging, brain images, sparse representation, metric learning

## Abstract

Magnetic resonance imaging (MRI) can have a good diagnostic function for important organs and parts of the body. MRI technology has become a common and important disease detection technology. At the same time, medical imaging data is increasing at an explosive rate. Retrieving similar medical images from a huge database is of great significance to doctors’ auxiliary diagnosis and treatment. In this paper, combining the advantages of sparse representation and metric learning, a sparse representation-based discriminative metric learning (SRDML) approach is proposed for medical image retrieval of brain MRI. The SRDML approach uses a sparse representation framework to learn robust feature representation of brain MRI, and uses metric learning to project new features into the metric space with matching discrimination. In such a metric space, the optimal similarity measure is obtained by using the local constraints of atoms and the pairwise constraints of coding coefficients, so that the distance between similar images is less than the given threshold, and the distance between dissimilar images is greater than another given threshold. The experiments are designed and tested on the brain MRI dataset created by Chang. Experimental results show that the SRDML approach can obtain satisfactory retrieval performance and achieve accurate brain MRI image retrieval.

## Introduction

The number of tumor patients has increased rapidly in recent years. Tumors have become one of the most common diseases in the world. The prevention, early diagnosis, and treatment of tumors have aroused widespread concern and discussion in the medical community and the public. Pathological examination is still the gold standard for tumor detection in the medical field. Medical imaging is a common method for diagnosing tumors, such as X-ray, magnetic resonance imaging (MRI), and computed tomography (CT) scans (Zhang et al., 2019; Chahal et al., 2020; Jiang et al., 2021). At present, early cancer screening mainly depends on the experience of doctors. The accuracy of decision-making largely depends on the knowledge and experience of doctors and the quality and quantity of information available. At the same time, the development of digital imaging technology and artificial intelligence has jointly promoted the emergence of digital pathology. Digital pathology aims to use computers to automatically analyze the characteristics of digital histopathology images to serve different tasks such as detection, segmentation, and retrieval. Digital pathology can provide physicians with knowledge-supported assistive technology in their decision-making process, which is of great practical significance.

With the popularization of Internet technology, the scale of medical imaging databases is getting larger and larger, and more and more past cases can be provided to doctors. Content-based image retrieval (CBIR) has technical advantages in medical diagnosis (Müller and Unay, 2017; Zheng et al., 2018). CBIR not only provides doctors with a prediction, but also improves the efficiency and accuracy of diagnosis by retrieving similar historical cases. Therefore, CBIR is essentially consistent with the physician’s diagnostic decision-making process. In addition, the use of CBIR can develop knowledge-based medical diagnosis decision support systems and case interpretation systems. Medical image retrieval is the core technology of the CBIR system, which can retrieve medical images similar to the image to be diagnosed from the medical database. We know that medical images are very different from natural images. First, the resolution of medical images is relatively high, but most of them are grayscale images. Secondly, the important information of medical images is mostly concentrated in small areas. Thirdly, the semantic content of visually similar medical images may be very different. Therefore, the retrieval effect is often unsatisfactory when the conventional natural image processing method is directly applied to medical imaging. Especially for brain tumor images, the shape, size, and texture of the same type of brain tumor are different due to the severity of the patient’s condition, age, and other factors. Therefore, different pathological types may show similar appearances, which make the recognition of brain tumor images very challenging.

Medical image retrieval is to retrieve images similar to the histopathological images to be diagnosed from the database, to provide references for doctors’ diagnoses. The histopathological image retrieval algorithm generally includes two stages: feature extraction and similarity measurement model construction (Zhang et al., 2015). The tumor boundary in brain tumor images is usually unclear, so the shape information cannot provide a clear tumor contour area. Therefore, it is important to determine the intensity and texture features. Huang et al. (2012) combined the spatial pyramid model and the Bow model and used the bag-of-words (BoW) histogram with spatial information to describe the characteristics of brain tumor regions, which improved the retrieval performance of brain tumor images. Huang et al. (2014) regarded the boundary of the brain tumor as the region of interest (ROI), used the region division learning method, and expressed the features with the original image pixel intensity. Finally, the authors aggregated the local features of each subregion and spatialized them, to improve the discriminative ability of image features. Cheng et al. (2016) adopted adaptive space pool and fisher vector method for brain tumor image retrieval.

In the field of medical image retrieval, similarity measurement refers to using a given medical image pair to calculate the distance between the feature vectors of medical image pairs, and it is often used to judge the type of medical image. For medical images, similarity measures include semantic correlation and visual similarity. The semantic correlation measure depends on the class labels of medical images. For example, the labels of two brain MRI images are healthy, which means that the two brain MRI images are semantically related. The visual similarity measure describes the similarity of features, which usually describes the similarity of medical images from a visual perspective. The definition of similarity measures generally considers the use of distance measures. The commonly used distance measurement methods include Euclidean distance, angle cosine distance, Mahalanobis distance, and Minkowski distance, etc., Yang et al. (2012) extracted local features based on pixel intensity information along the brain tumor boundary and used BoW to generate global features. At the same time, the authors designed a distance metric learning method to improve retrieval accuracy. Verma and Jawahar (2017) proposed a two-pass k-nearest neighbor model. Firstly, a more balanced training data set was designed for each image to reduce the label imbalance problem. Then, the large interval nearest neighbor algorithm was extended to the case of multi-label classification for metric learning, and the optimal weights of combined basis distance and features were obtained for semantic label prediction. Gu et al. (2017) proposed a distance measurement method of weighted heterogeneous values for tumor diagnosis.

Inspired by the sparse mechanism of the human visual system, sparse representation has been widely used in the field of computer vision. Sparse representation succinctly represents the image to be labeled as a linear combination of a few atoms in the dictionary. These sparse representation coefficients and corresponding atoms fully reveal the internal essence of the image and also adapted to human visual perception. Dictionary learning is a common method in sparse representation research. The dictionary learned from a large number of samples can adaptively sparse represent various feature information in the image, which greatly improves the sparse representation performance. Sparse representation methods can be divided into two categories. The first is methods based on predefined dictionaries. Srinivas et al. (2014) used the correlation between the RGB three-channels of color images to construct a dictionary and applied it to histopathological image representation and classification. However, this method based on the predefined dictionary may not be able to make full use of the discriminative information hidden in the training samples. The second type of method learns the dictionaries from training samples. For example, Gao et al. (2014) regarded the samples of each subclass as a subgroup and used the multi-layer group sparsity to classify and annotate images. Tang et al. proposed a semi-supervised learning method based on a sparse graph for image classification. By using the multi-scale and spatial information of the image, Tang et al. (2010) proposed a multi-scale representation learning algorithm for breast image classification. Zhang et al. (2015) proposed a heterogeneous feature fusion method and applied it to the image classification of pathological tissues. The above methods mainly focus on the reconstruction of histopathological images, so their classification performances are not optimal. The dictionary learning method based on supervised learning uses robust feature representation from the original visual information through mining supervised or semi-supervised features and coding coefficients. Cao et al. (2015) used the group sparse reconstruction method to learn the semantic correlation of symbiotic labels for optimizing the dictionary. The authors used the group sparse framework to reconstruct the test image for label prediction and realized weakly supervised image annotation. Lu et al. (2015) studied the semantic sparse recording method of visual content to generate a more descriptive and robust bow representation for image classification.

This paper proposes sparse representation-based discriminative metric learning (SRDML) approach for brain MRI image retrieval. SRDML integrates sparse representation and metric learning into a discriminative model. On the one hand, the model uses the sparse representation to learn the robust feature representation of brain MRI images. On the other hand, the model learns a metric space with discriminative ability, so that similar brain MRI images are closely projected in the metric space, and dissimilar brain MRI images are separated from each other as much as possible. The advantages of SRDML are as follows: (1) the local information retention term of coding coefficients maintains the semantic correlation and visual similarity of brain MRI images in the projection space. (2) Our approach finds an appropriate metric matrix under the constraints of similarity, so that the distance between similar images’ coding coefficients is less than the given threshold, and the distance between dissimilar images’ coding coefficients is greater than the given threshold. Its goal is to achieve the dispersion of “maximum inter-class” and “minimum intra-class”. (3) The learning of sparse representation and metric learning is optimized through alternate iterations. This learning strategy allows the model parameters to reach the optimal solution at the same time. (4) The experimental results show that SRDML has achieved good performance on the brain MRI image dataset, which shows the feasibility of our retrieval approach in the brain tumors diagnosis.

The rest of this paper is organized as follows: first, related works of metric learning and sparse representation are introduced. Second, the proposed sparse representation-based discriminative metric learning approach is formulated in section “Sparse Representation-Based Discriminative Metric Learning”. Third, experiments are presented to verify our approach. Finally, a summary of our approach is presented, and its future work is discussed.

## Backgrounds

### Metric Learning

The traditional metric learning is based on labeled samples of medical image data sets and the label information appears in the form of paired constraints of samples. Assuming that the medical image data set is expressed as ${\left\{x_{i}\right\}}_{i=1}^{N}\in R^{d}$, where *x*_*i*_ is the *i*th sample, taking Mahalanobis distance as an example, the distance measurement between two images *x*_*i*_ and *x*_*j*_ can be written as,


(1) $$d_{M}^{2}\,\left(x_{i},x_{j}\right)={||x_{i}-x_{j}||}_{M}^{2}={\left(x_{i}-x_{j}\right)}^{T}\,M\,\left(x_{i}-x_{j}\right).$$

The positive semi-definite matrix **M** can be decomposed into *M* = *W^T^W*, and the matrix *W* ∈ *R^d^*^×*m*^(*m*≤*d*)is called the metric matrix. Therefore, Eq. 1 can be expressed as,


(2) $$d_{M}^{2}\,\left(x_{i},x_{j}\right)={\left({\mathrm{Wx}}_{i}-{\mathrm{Wx}}_{j}\right)}^{T}\,\left({\mathrm{Wx}}_{i}-{\mathrm{Wx}}_{j}\right).$$

Therefore, the essence of the Mahalanobis metric is to learn a projection space, in which that similar image pairs output a positive value close to zero, and dissimilar image pairs output a larger value.

A type of famous metric learning algorithm is large-margin distance metric learning (Weinberger and Saul, 2009; Nguyen et al., 2020). Let **S** and **D** be two datasets of pairwise constraints: **S** = {(*x*_*i*_, *x*_*j*_) | *x*_*i*_ and *x*_*j*_ are similar}, and **D** = {(*x*_*i*_, *x*_*j*_) | *x*_*i*_ and *x*_*j*_ are dissimilar}. The large-margin distance metric learning takes the paired similar or dissimilar data sets as model input and constructs the metric learning as a convex optimization problem,


(3) $$\begin{array}{ccc} \min_{M} & & \sum_{\left(x_{i}-x_{j}\right)\in S}{||x_{i}-x_{j}||}_{M}^{2},\\ s.t. & & \sum_{\left(x_{i}-x_{j}\right)\in D}{||x_{i}-x_{j}||}_{M}^{2}\geq c,\\ & & M\geq 0, \end{array}$$

where *c* is a positive. It can be seen from Eq. 3 that the distance between dissimilar pair samples is greater than the constant *c*, while the distance between similar pair samples is as small as possible.

### Sparse Representation

The goal of sparse representation is to use the linear combination of dictionary atoms to represent the observation data, and to use sparsity constraints on the combination coefficients, so that each observation data is only represented by a subset of all dictionary atoms (Gu et al., 2021a,b). The traditional sparse representation framework is,


(4) $$\begin{array}{ccc} \min_{B,Y} & & {||\text{X-BY}||}_{F}^{2}+\lambda \,\phi \,\left(Y\right),\\ s.t. & & {||b_{i}||}_{2}\leq 1,\forall i, \end{array}$$

where **B** and **Y** are the dictionary matrix and sparse coding coefficient matrix, respectively. $|||{|}_{F}^{2}$ is *F*-norm.

In order to avoid that the value of **B** is too large and the sparse coefficient **Y** is too small, the dictionary column vector is usually set to satisfy the constraint ||*b*_*i*_||_2_≤1. ϕ(*Y*) is the regularization term controlling the sparsity of **Y**. λ is the regularization parameter. The objective function is not a joint convex function of (**B**, **Y**), but when one variable is fixed, the objective function is convex for the other variable. Therefore, Eq. 4 can be solved by a turn-by-turn optimization method.

## Sparse Representation-Based Discriminative Metric Learning

In order to better explain the proposed model, first, we introduce some important terms: (1) a labeled training image set *X* = [*x*_1_,*x*_2_,…,*x*_*n*_] ∈ *R^d^*^×*n*^; (2) the testing image set contains images available for the retrieval of the target brain MRI images; (3) each image in testing image set is considered as the query image.

### The Pairwise Constraint of Sparse Coding Coefficients

From the perspective of image retrieval, similar images should have similar coding coefficients, and dissimilar images should have different coding coefficients. In other words, in the learned metric space, the smaller the better the distance between similar images, at the same time, the greater the better the distance between dissimilar images, to reduce the uncertainty of misjudging the coding coefficient of dissimilar images as similar images. Based on this idea, two thresholds σ_1_ and σ_2_ are set for calculating the metric distances of similar images and dissimilar images in the metric space as,


(5) $$\left\{ \begin{array}{c} d_{M}^{2}\,\left(y_{i}-y_{j}\right)<\sigma_{1},i\,f\,\left\{x_{i},x_{j}\right\}\in S\\ d_{M}^{2}\,\left(y_{i}-y_{j}\right)>\sigma_{2},i\,f\,\left\{x_{i},x_{j}\right\}\in D \end{array}\right.$$

where σ_1_ < σ_2_.

To unify the Eq. 5 into an inequality, a common threshold is introduced, i.e., σ_1_ = τ−1 and σ_2_ = τ + 1, then Eq. 5 can be written as,


(6) $$\ell_{i\,j}\,\left(\tau -d_{M}^{2}\,\left(y_{i}-y_{j}\right)\right)>1,$$

where if *x*_*i*_ and *x*_*j*_ are similar images, ℓ_*ij*_ = 1; otherwise, *x*_*i*_ and *x*_*j*_ are dissimilar images, ℓ=i⁢j-1.

With the pairwise constraint of coding coefficients, it is expected to have a large margin between each similar and dissimilar pairs in the learned metric space. By introducing the generalized logistic loss function $g\,\left(y\right)=\frac{1}{\theta }\,\log\left(1+\exp\left(\theta \,y\right)\right)$ with the sharpness parameter θ, the pairwise constraint of coding coefficients can be defined as,


(7) $$\min_{Y}g\,\left(1-\ell_{i\,j}\,\left(\tau -d_{M}^{2}\,\left(y_{i}-y_{j}\right)\right)\right).$$

### The Locality Constraint of Atoms

First, to exploit the local structure information in the sparse representation learning, the local manifold of the dictionary atoms is built on the nearest neighbor graph **P** of dictionary **B** ∈ *R^d^*^×*K*^. The element *P*_*ij*_ of **P** is defined as,


(8) $$P_{i\,j}=\left\{ \begin{array}{cc} \text{exp}\,\left(-\frac{{||b_{i}-b_{j}||}_{2}^{2}}{\mu }\right), & i\,f\,b_{j}\in \text{KNN}\,\left(b_{i}\right)\\ 0, & e\,l\,s\,e \end{array}\right.$$

where KNN() is the *k*-nearest neighbor function, and μ is the KNN parameter.

In the learned metric space, we assume that the similar images will keep close to each other, thus it is expected the similar dictionary atoms will keep close to each other as well. A Laplacian matrix **L** is constructed based on the matrix **P**,


(9) L=W-P,

where *W* = *diag*(*W*_1_,…,*W*_*K*_), and $W_{i}=\sum_{j}^{K}P_{i\,j}$. *K* is the number of atoms in **B**.

Using the Laplacian matrix **L**, the locality constraint of atoms is represented as,


(10) $$\min_{Y}\frac{1}{2}\,\sum_{i}^{K}\sum_{j}^{K}{\left(y_{i}-y_{j}\right)}^{2}\,P_{i\,j}=T\,r\,\left(Y^{T}\,L\,Y\right).$$

### The Objective Function of SRDML and Its Optimization

Now we have the two terms needed to be embedded into the sparse representation framework and compose the SRDML objective function. SRDML combines the sparse representation, pairwise constraint of coding coefficients, and locality constraint of atoms together to yield an image retrieval approach. The SRDML approach takes advantage of pairwise metric constraints and local structure knowledge preserved provided by the coding coefficients. The objective function of SRDML is,


(11) $$\begin{array}{ccc} \min_{M,B,Y} & & {||\text{X-BY}||}_{2}^{2}+\alpha \,T\,r\,\left(Y\,L\,Y\right)+\beta \,\sum_{i,j}g\,\left(1-\ell_{i\,j}\,\left(\tau -d_{M}^{2}\,\left(y_{i}-y_{j}\right)\right)\right)\\ & & +\gamma \,{||Y||}_{2}^{2},\\ s.t. & & M\geq 0,\\ & & {||b_{i}||}^{2}=1,\forall i \end{array}$$

where α, β, and γ are regularization parameters.

There are three variables {M,B,Y} needed to be tuned in the SRDML approach. An alternating optimization method is used to solve Eq. 11.

Tune **B**. With fixed **M** and **Y**, the objective function of **B** can be written as,


(12) $$\begin{array}{ccc} \min_{B} & & {||\text{X-BY}||}_{2}^{2},\\ s.t. & & {||b_{i}||}^{2}=1,\forall i, \end{array}$$

Obviously, it is a least square problem with quadratic constrains. The Lagrange dual function is used, and we have,


(13) $$h\,\left(B,\tau \right)=\left({||\text{X-BY}||}_{2}^{2}+\sum_{i}^{K}\tau_{i}\,\left({||b_{i}||}^{2}-1\right)\right),$$

where τ is the Lagrange vector. A diagonal matrix Δ ∈ *R^K^*^×*K*^ is introduced, where Δ_*ii*_ = τ_*i*_ for all *i*. We can obtain the following problem,


(14) $$h\,\left(B,\tau \right)=\left({||\text{X-BY}||}_{2}^{2}+T\,r\,\left(B^{T}\,B\,\Delta \right)-T\,r\,\left(\Delta \right)\right).$$

Taking the first-order partial derivatives of **B** in Eq. 14, we can obtain the close-solution of **B** as,


(15) $$B^{*}=X\,Y^{T}\,{\left(Y\,Y^{T}+\Delta \right)}^{-1}.$$

Tune **Y**. With fixed **M** and **B**, the objective function of **Y** can be written as,


(16) $$\min_{Y}{||\text{X-BY}||}_{2}^{2}+\alpha \,T\,r\,\left(\text{YLY}\right)+\beta \,\sum_{i,j}g\,\left(1-\ell_{i\,j}\,\left(\tau -d_{M}^{2}\,\left(y_{i}-y_{j}\right)\right)\right)+\gamma \,{||Y||}_{2}^{2},$$

Taking the first-order partial derivatives of *y*_*i*_ in Eq. 16, we can obtain the close-solution of *y*_*i*_ as,


(17) $$y_{i}^{*}={\left(B^{T}B+\gamma I+\alpha L+\beta \sum_{i,j}g^{\prime }\left(\Lambda \right)M\right)}^{-1}(B^{T}Y+\beta M\sum g^{\prime }\left(\Lambda \right)y_{j}),$$

where $\Lambda =1-\ell_{i\,j}\,\left(\tau -d_{M}^{2}\,\left(y_{i}-y_{j}\right)\right)$.

Tune **M**. With fixed **Y** and **B**, the objective function of **M** can be written as,


(18) $$\begin{array}{c} \min_{M}\beta \,\sum_{i,j}g\,\left(1-\ell_{i\,j}\,\left(\tau -d_{M}^{2}\,\left(y_{i}-y_{j}\right)\right)\right),\\ s.t.M\geq 0, \end{array}$$

The Lagrange dual function is used, and we have,


(19) $$h\,\left(M,\lambda \right)=\beta \,\sum_{i,j}g\,\left(1-\ell_{i\,j}\,\left(\tau -d_{M}^{2}\,\left(y_{i}-y_{j}\right)\right)\right)+\lambda \,{||\text{M-I}||}_{2}^{2},$$

where λ is the Lagrange parameter.

Let *s*_*ij*_ = (*y_i_*−*y_j_*)*^T^*(*y*_*i*_−*y*_*j*_), taking the first-order partial derivatives of **M** in Eq. 19, we obtain,


(20) $$\frac{\partial h}{\partial M}=\beta \,\sum_{\left\{x_{i},x_{j}\right\}\in A_{s\,e\,t}}g^{\prime }\,\left(\Lambda \right)\,C_{i\,j}+2\,\gamma \,\left(M-I\right).$$

Then we can obtain *M* by $M_{t}=M_{t-1}-\xi \,\frac{\partial h}{\partial M_{t-1}}$, where ξ is the learning rate in gradient descent method.

The proposed SRDML approach is shown in algorithm 1.

Algorithm 1 SRDML approach

Input: Similar image pair subset and dissimilar image pair subset in **X**, parameters α, β, and γ;

Output: coding coefficient matrix **Y**, dictionary **B**, and metric matrix **M**;

1: Initializing **B** and **Y** with the K-SVD algorithm, **M** with the identity matrix;

2: Calculating graph Laplacian matrix **L** using Eqs 8 and 9;

3: while *t*≤ maximum number of iterations *T*_*max*_

Calculating dictionary **B** using Eq. 15;
Calculating graph Laplacian matrix **L** using Eqs 8 and 9;
Calculating coding coefficient matrix **Y** using Eq. 17;
Calculating metric matrix **M** using Eq. 20;

4: Obtaining B* = *B^T^_max_*, Y* = *Y^T^_max_*, and M* = *M^T^_max_*.

## Experiments

### Experimental Settings

**FIGURE 1:**
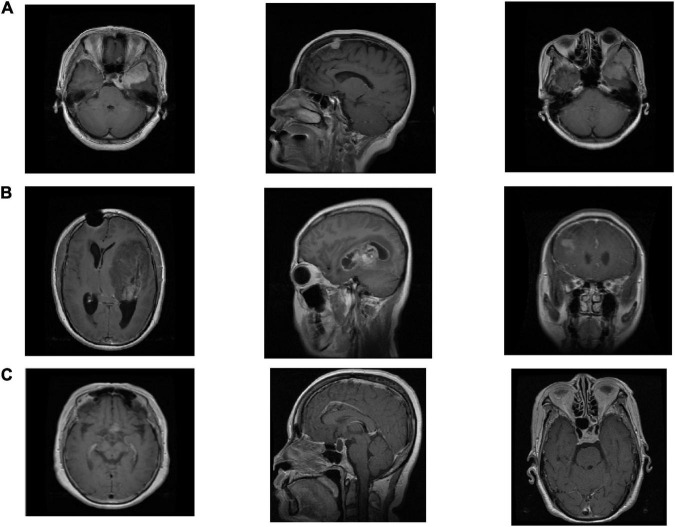
The example samples in the Cheng dataset, **(A)** Meningioma, **(B)** Glioma, and **(C)** Pituitary.

We use the Cheng brain MRI dataset (Cheng et al., 2016) to evaluate the effectiveness of the proposed SRDML approach. This brain MRI dataset contains a total of 3064 slices from 233 patients. The brain MRI images contain three types of tumor images Meningioma, Glioma, and Pituitary. The numbers of the three types of tumor images are 708, 1426, and 930, respectively. The size of each image is 512 × 512 pixels. 600 images of each type of brain tumor are selected in the experiment. Figure 1 depicts the example samples of the Cheng dataset.

We use the GIST descriptor implemented by Gumaei et al. (2018, 2019) in this study. GIST descriptor extracts the image features based on the spatial envelope. In the experiment, we obtain 512 dimensional features of each image. We randomly divide brain MRI images into five subgroups of the same size. It is ensured that the different types of tumors in the five subgroups do not overlap and had equal proportions. We use 5-fold cross-validation to evaluate performance. Each image in the test data set is regarded as a query image. We adopt the mean average precision (*mAP*) and top-n retrieval precision (named as Prec@n) (Cheng et al., 2016). *mAP* and Prec@n can be calculated as


(21) $$m\,A\,P=\frac{\sum_{i=1}^{n}\frac{i}{r\,a\,n\,k\,\left(i\right)}}{n},$$

where *n* represents the number of retrieved images of the same type of brain tumor in the dataset, *rank*(*i*) represents the ranking number of the *i*-th retrieved image of the same type of brain tumor in the search results.


(22) $$P\,r\,e\,c\,@\,n=\frac{1}{s}\,\sum_{i=1}^{s}\sum_{j=1}^{k}\frac{j}{P\,o\,s\,i\,t\,i\,o\,n\,\left(j\right)},$$

where *s* is the number of queries, and *Position*(*j*) refers to the position of the *j*-th relevant sample in the search results.

The retrieval performance of SRDML is compared with various matrix learning algorithms LMNN (Weinberger and Saul, 2009), RCA (Bar-Hillel et al., 2005), MPP (Yang et al., 2012), TFFHD (Sun et al., 2018), and RDML-CCPVL (Ni et al., 2018). The parameter setting of the comparison algorithm refers to the setting of the original literature. The parameters of SRDML are set as follows. The size of the dictionary is equal to the number of the training set. The regularization parameters are set in the grid {10^−3^,10^−2^,…,10}. The dimension of metric matrix is set in the grid {50,100,200,…,500}. The sharpness parameter in generalized logistic loss function is set to be 1. The KNN parameter is set in the grid {3,5,…,13}. The running environment of all algorithms is CPU i7-8700k, 3.2 GHZ, 32GB RAM, and the running software is Matlab 2019.

### Performance Evaluation

**FIGURE 2:**
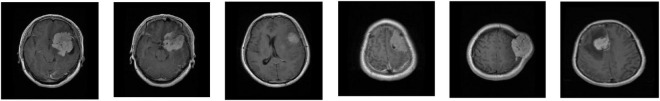
Retrieval result examples of SRDML in Meningioma.

**FIGURE 3:**
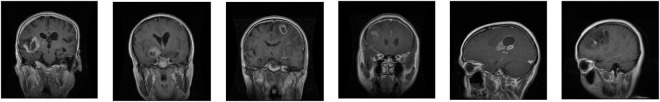
Retrieval result examples of SRDML in Glioma.

**FIGURE 4:**
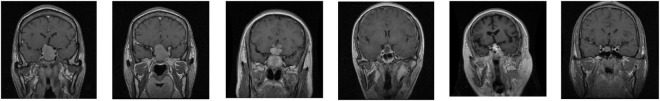
Retrieval result examples of SRDML in Pituitary.

Tables 1–3 list the retrieval performance of SRDML for the query image Meningioma, Glioma, Pituitary, respectively. These tables show the results of the 5-fold cross validation test set. We can see that the results obtained by the SRDML approach are consistent on the 5-fold test set, which verifies that the retrieval performance of our approach is stable. We conduct further case analysis on the SRDML. Figures 2–4 depict the retrieval result examples of SRDML in Meningioma, Glioma, Pituitary, respectively. The first column of images in Figures 2–4 are the query images. The second to sixth images are five retrieved images. We can see that the retrieved images are all highly related to the query images. The results show that SRDML can give full play to its advantages in brain MRI sets. The SRDML approach uses sparse representation and metric learning to mine the structural features and discrimination information of the data. The local information term and similarity constraints based on coding coefficients ensure the “maximum inter-class dispersion” and “minimum intra-class dispersion” of the extracted brain MRI features. Thus, the SRDML approach has the important practical value in assisting clinical diagnosis.

**TABLE 1:** Retrieval performance of SRDML for the query image Meningioma on 5-fold test set.

	*m*AP	prec@10	prec@20
Fold 1	92.12	89.93	89.93
Fold 2	92.13	89.95	89.93
Fold 3	92.14	89.92	89.88
Fold 4	92.19	89.97	89.96
Fold 5	92.19	89.95	89.92
Mean (standard deviation)	92.15 (0.030)	89.94 (0.017)	89.92 (0.026)

**TABLE 2:** Retrieval performance of SRDML for the query image Glioma on 5-fold test set.

	*m*AP	prec@10	prec@20
Fold 1	97.87	95.75	95.74
Fold 2	97.86	95.71	95.72
Fold 3	97.81	95.68	95.70
Fold 4	97.80	95.70	95.70
Fold 5	97.75	95.68	95.67
Mean (standard deviation)	97.82 (0.044)	95.70 (0.026)	95.70 (0.023)

**TABLE 3:** Retrieval performance of SRDML for the query image Pituitary on 5-fold test set.

	*m*AP	prec@10	prec@20
Fold 1	97.87	96.45	96.44
Fold 2	97.93	96.43	96.46
Fold 3	97.94	96.50	96.50
Fold 4	97.96	96.51	96.47
Fold 5	97.87	96.46	96.54
Mean (standard deviation)	97.92 (0.037)	96.47 (0.030)	96.48 (0.035)

### Performance Comparison

**FIGURE 5:**
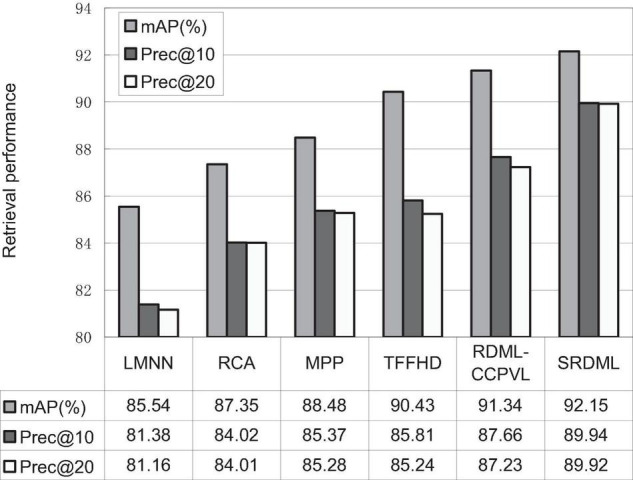
Retrieval performance comparisons in Meningioma.

**FIGURE 6:**
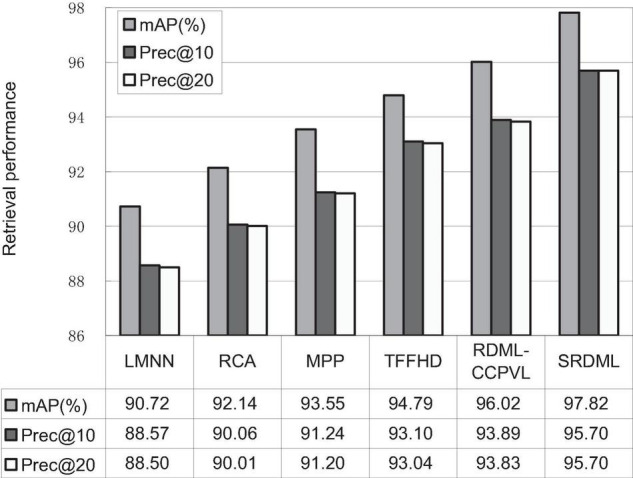
Retrieval performance comparisons in Glioma.

**FIGURE 7:**
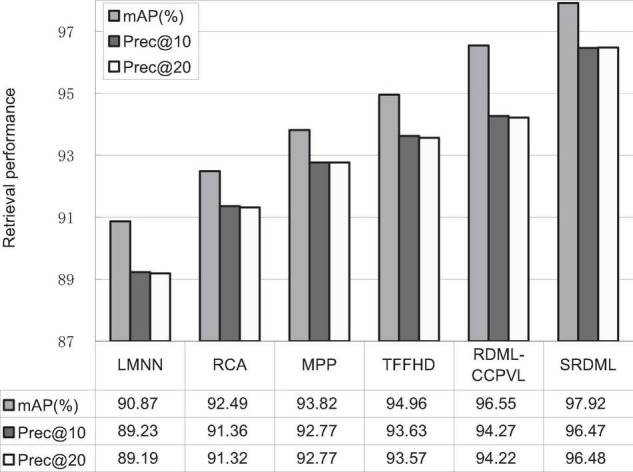
Retrieval performance comparisons in Pituitary.

We use the SRDML approach to compare the performance with LMNN, RCA, MPP, TFFHD, and RDML-CCPVL. Figures 5–7 show the retrieval performance of various comparison algorithms in Meningioma, Glioma, and Pituitary. The results show these methods in retrieval performance of *m*AP, prec@10, as well as Prec@20. According to the results in Figures 5–7, the following results are obtained:

(1) In the Meningioma, the SRDML approach is compared with the second-best in *m*AP, prec@10, as well as prec@20 by 0.81, 1.28, and 2.69%, respectively. SRDML has excellent retrieval performance, especially in Prec@20. This shows that the proposed approach has high retrieval performance. In the Glioma, the SRDML approach is compared with the second-best approach in *m*AP, prec@10 as well as prec@20 increased by 1.80, 1.81, and 1.87%, respectively. In the Pituitary, the SRDML approach is compared with the second-best approach in *m*AP, Prec@10, as well as Prec@20 increased by 1.37, 2.20, and 2.26%, respectively.
(2) The SRDML approach has significantly improved the retrieval performance compared with the traditional approaches. The SRDML approach uses the sparse representation to learn the robust feature representation of brain MRI images, and learns the metric space with discrimination ability, so that the similar brain MRI pairs are closely mapped in the metric space and the dissimilar brain MRI pairs are separated from each other as much as possible.

### Parameter Analysis

The parameters of the SRDML approach are obtained by the grid optimization method. Here we mainly evaluate the influence of KNN parameter *k* and the dimension of metric matrix *m*. Table 4 lists the *m*AP(%) of the SRDML approach with different *k.* The parameter *k* is set in the grid {3,5,…,13}. The value of *k* increases from 3 to 7, and the *m*AP value increases significantly. It is verified that the local information item is an important part in the SRDML approach. Extracting the appropriate local information can be helpful to describe the image features and improve retrieval accuracy. When the value of *k* increases from 9 to 13, the retrieval performance begins to decrease, indicating that too large the value of *k* will cause useless information of the local information and too much useless information, which affects the retrieval performance of SRDML. Table 5 lists the *m*AP(%) of the SRDML approach with different *m.* The parameter *m* is set in the grid {50,100,200,…,500}. It can be seen from the results in Table 5 that when *m* is set as 100 or 200, *m*AP(%) of the SRDML approach can reach the optimal retrieval performance. If the value of *m* is too large or too small, the metric space cannot properly represent the internal data structure of brain MRI images. Therefore, it is feasible to use the grid search method to determine the optimal values of *k* and *m*.

**TABLE 4:** *m*AP(%) of the SRDML approach with different *k*.

	*k* = 3	*k* = 5	*k* = 7	*k* = 9	*k* = 11	*k* = 13
Meningioma	90.53	91.77	**92.15**	**92.15**	92.00	91.42
Glioma	96.50	97.08	97.69	**97.82**	97.47	96.99
Pituitary	97.09	97.51	**97.92**	97.89	97.44	97.01

*The bold values represent the best results in comparison experiments.*

**TABLE 5:** *m*AP(%) of the SRDML approach with different *m*.

	*m* = 50	*m* = 100	*m* = 200	*m* = 300	*m* = 400	*m* = 500
Meningioma	89.98	91.43	**92.15**	92.10	92.12	91.58
Glioma	96.90	**97.82**	**97.82**	97.59	97.60	97.01
Pituitary	96.86	**97.92**	97.90	97.80	97.33	97.02

*The bold values represent the best results in comparison experiments.*

## Conclusion

The purpose of medical image retrieval is to retrieve similar image data from a huge imaging database. The retrieval results should not only be similar in image feature measurement, but also fit in image semantics as much as possible, to help doctors retrieve images with the same pathology. This paper proposes a similarity retrieval approach for brain MRI images based on sparse representation and metric learning. The characteristics of this approach are using sparse representation to extract the robust features of brain MRI image, which can more effectively represent the information of brain MRI image. The relative similarity constraint and local preserving information are used to project the low dimensional metric matrix, which improves the retrieval accuracy. The experiments are carried out on public data sets. The experiments show the effectiveness and accuracy of our approach in brain MRI image retrieval. The future work will be considered from the following aspects: (1) while improving the retrieval accuracy of brain MRI images, ensure the retrieval speed, to further provide assistance and support for doctors in diagnosing brain tumor lesions. (2) The experimental data scale of this paper is not large enough. During the retrieval process, several images with the highest similarity are returned by comparing the feature vector similarity of the data set in turn. When dealing with large-scale data, we will consider how to combine the technologies such as hash sorting of retrieval results to realize the real-time performance of retrieval results. (3) For different images and images with different principles, the feature algorithms involved are different. Forming a more general medical image retrieval system is the next research direction.

## Data Availability Statement

Publicly available datasets were analyzed in this study. This data can be found here: The Cheng dataset analyzed for this study can be found in this link [https://figshare.com/articles/dataset/brain_tumor_dataset/1512427].

## Author Contributions

GZ and TN conceived and designed the proposed model and wrote the manuscript. BL and XH ran the experiment and analyzed the results. All authors read and approved the manuscript.

## Conflict of Interest

The authors declare that the research was conducted in the absence of any commercial or financial relationships that could be construed as a potential conflict of interest.

## Publisher’s Note

All claims expressed in this article are solely those of the authors and do not necessarily represent those of their affiliated organizations, or those of the publisher, the editors and the reviewers. Any product that may be evaluated in this article, or claim that may be made by its manufacturer, is not guaranteed or endorsed by the publisher.
